# Supplementary material for: Haplotype Analysis of the Pre-harvest Sprouting Resistance Locus Phs-A1 Reveals a Causal Role of TaMKK3-A in Global Germplasm
Source: Front Plant Sci. 2017 Sep 13;8:1555. doi: 10.3389/fpls.2017.01555 (PMC5602128; doi:10.3389/fpls.2017.01555)
Supplement: Supplementary file 2 [file Image_1.PDF]

Association mapping and haplotype analysis of the pre-harvest sprouting resistance locus *Phs-A1* reveals a causal role of *TaMKK3-A* in global germplasm

Oluwaseyi Shorinola, Barbara Balcárková, Jessica Hyles, Josquin F. G. Tibbits, Matthew J. Hayden, Katarina Holušova, Miroslav Valárik, Assaf Distelfeld, Atsushi Torada, Jose M. Barrero and Cristobal Uauy*

*Corresponding author: Cristobal Uauy,
cristobal.uauy@jic.ac.uk ,
Phone: +44-(0)1603-450195

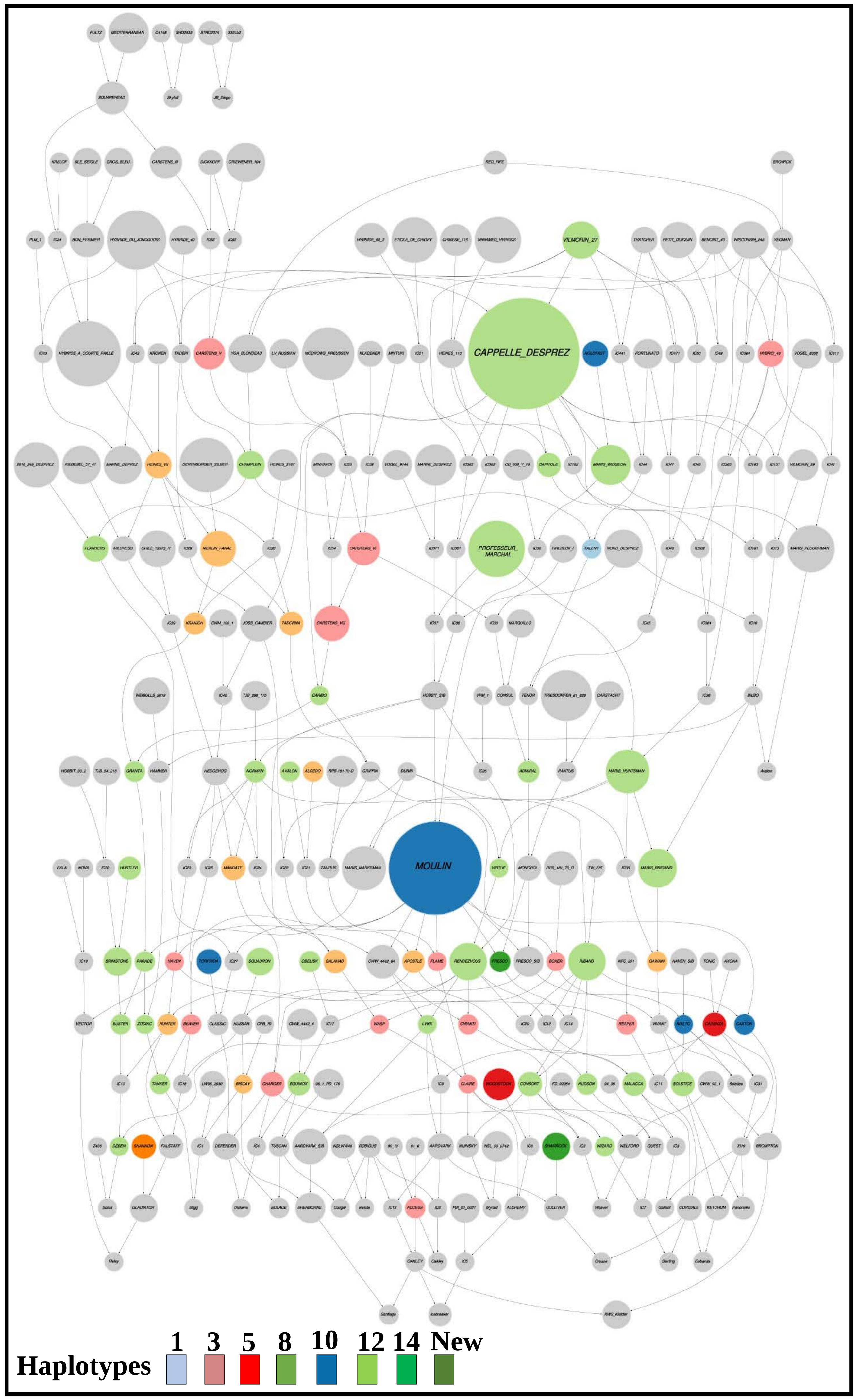

Figure S1: Pedigree of UK and European varieties with their corresponding *Phs-A1* haplotype status. Each circle represents a variety and the colours represent the different haplotypes.

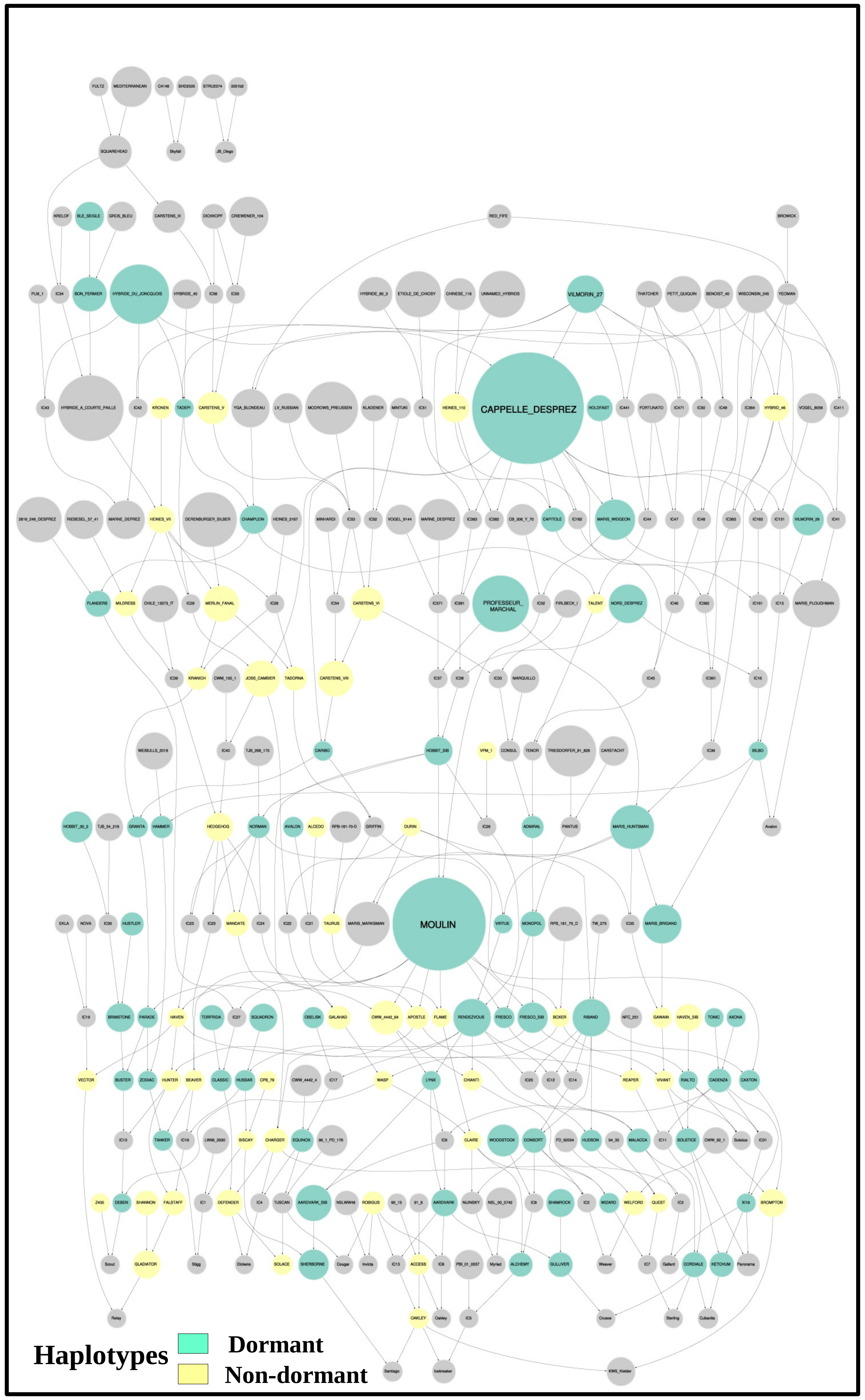

Figure S2: Pedigree of UK and European varieties with their corresponding *TaMKK3-A* allele. Each circle represents a variety and the colours represent the different haplotypes.

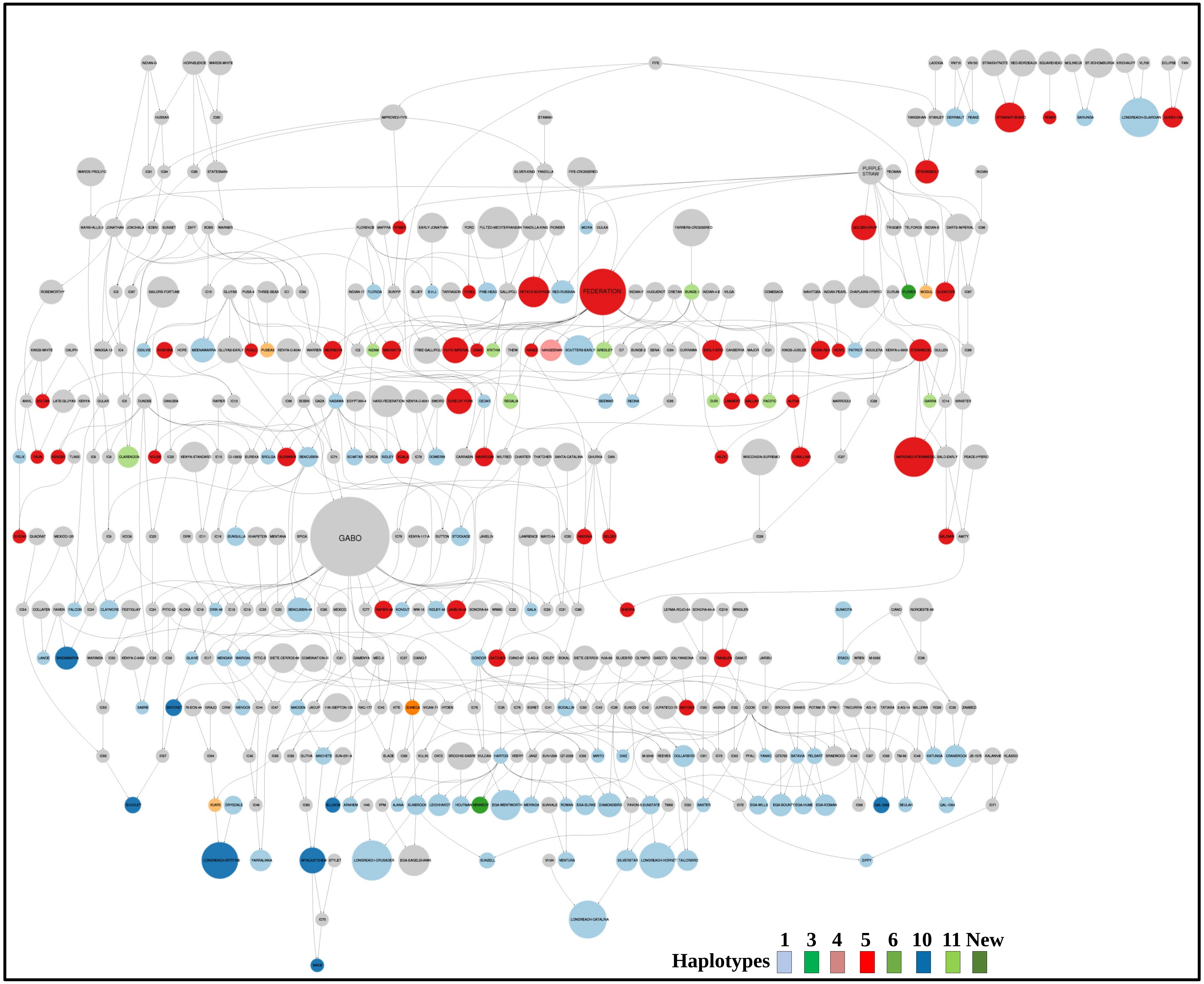

Figure S3: Pedigree of Australian varieties with their corresponding *Phs-A1* haplotype status. Each circle represents a variety and the colours represent the different haplotypes.

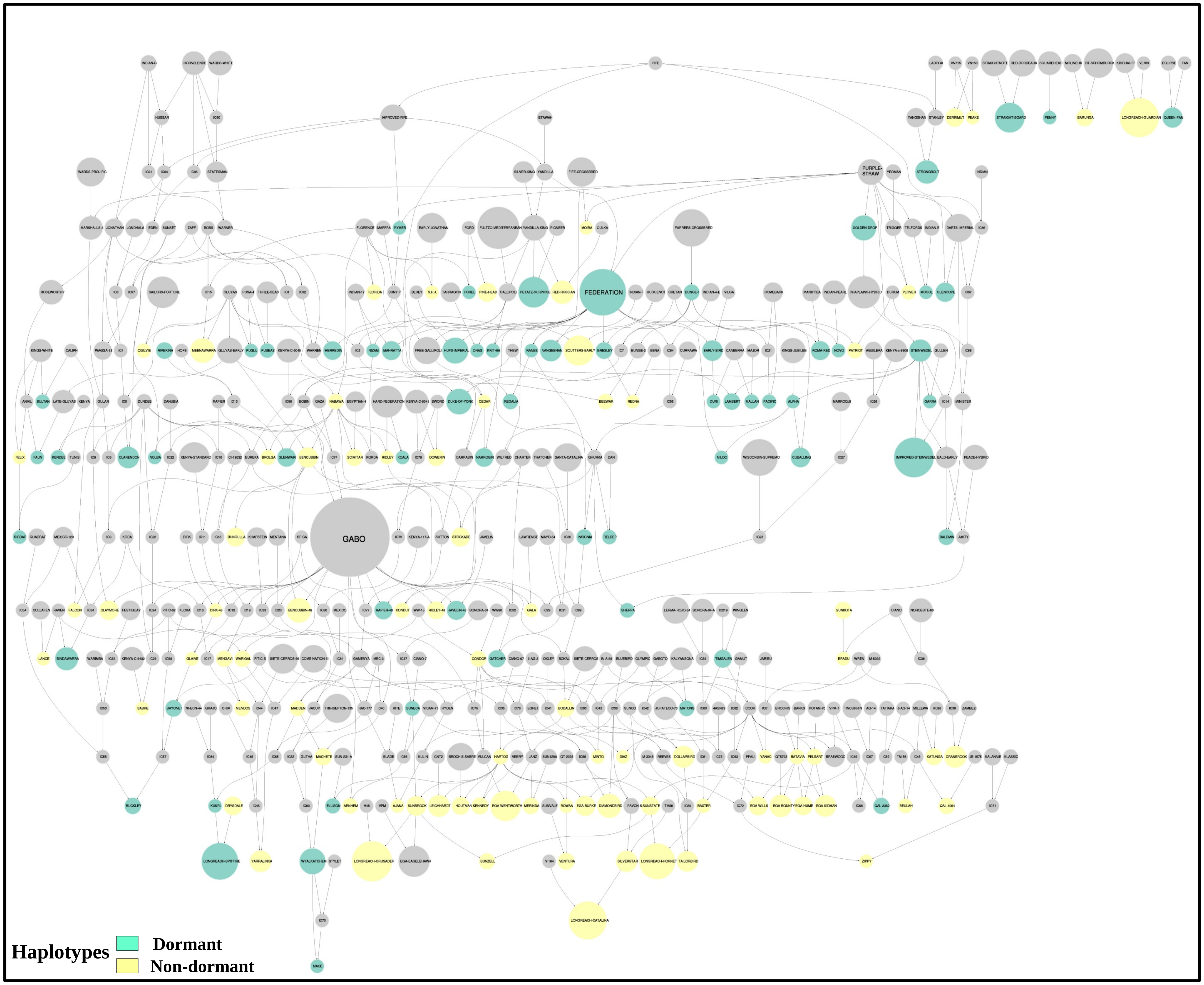

Figure S4: Pedigree of Australian varieties with their corresponding *TaMKK3-A* allele. Each circle represents a variety and the colours represent the different haplotypes.

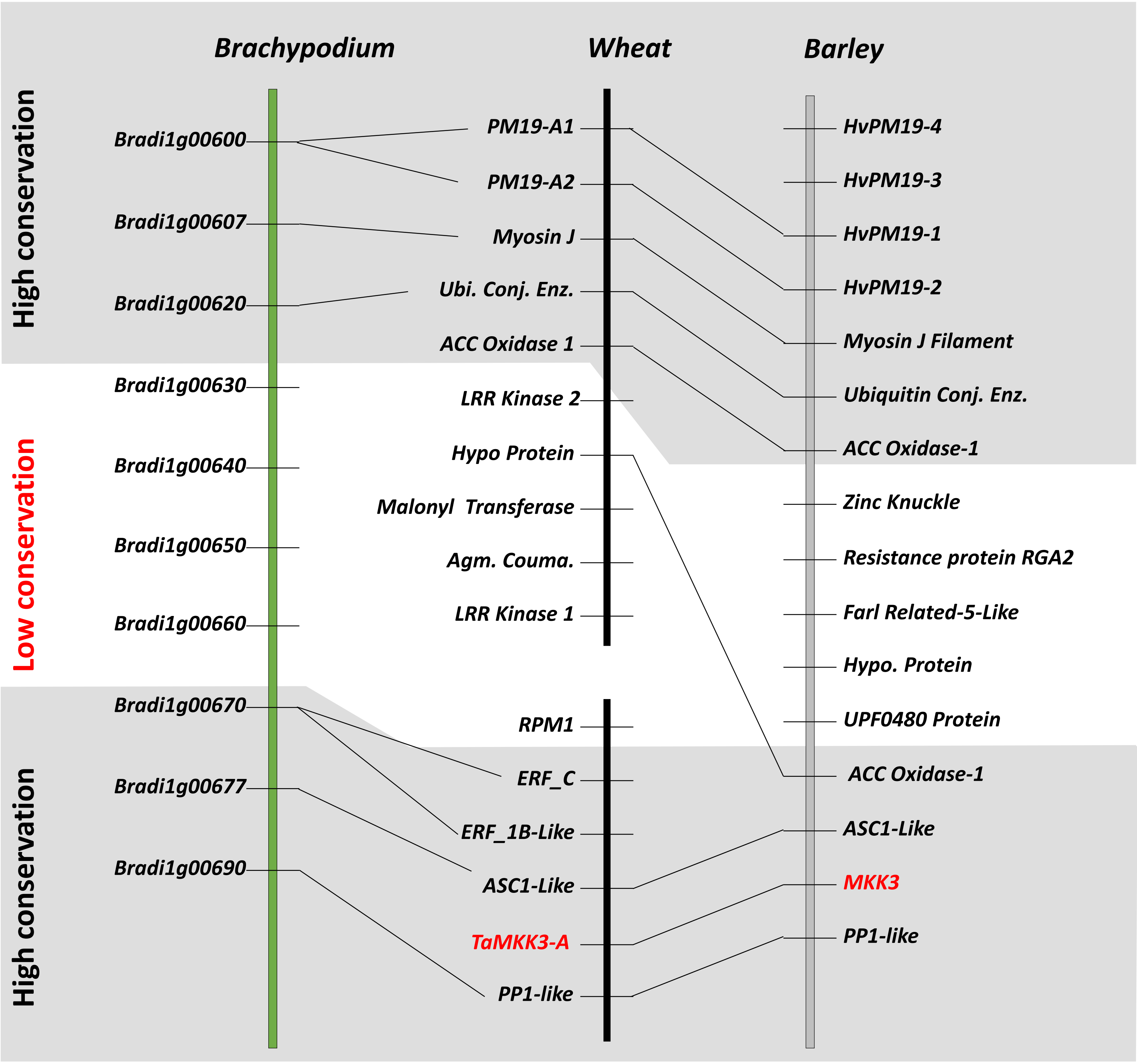

Figure S5: Comparison of syntenic *Phs-A1* physical maps and contigs in *Brachypodium*, wheat and barley. Genes in the homologous intervals in *Brachypodium* (amber line), wheat (black lines) and barley (grey line) are compared against each other. Orthologous genes across genomes are joined by lines. The region of high conservation is indicated with grey background while the region of low conservation has plain background.
